# Psoriasis and Hypertension Severity: Results from a Case-Control Study

**DOI:** 10.1371/journal.pone.0018227

**Published:** 2011-03-29

**Authors:** April W. Armstrong, Steven W. Lin, Cynthia J. Chambers, Mary E. Sockolov, David L. Chin

**Affiliations:** 1 Department of Dermatology, University of California Davis, Sacramento, California, United States of America; 2 Center for Healthcare Policy and Research, Graduate Group in Epidemiology, University of California Davis, Sacramento, California, United States of America; The University of Queensland, Australia

## Abstract

**Background:**

Epidemiologic studies have provided new insights into the association between psoriasis and cardiovascular diseases. Previous population studies have examined hypertension frequency in psoriasis patients. However, the relationship between severity of hypertension and psoriasis has not been characterized.

**Objective:**

We sought to investigate whether patients with psoriasis have more difficult-to-manage hypertension compared to non-psoriatic hypertensive patients.

**Approach:**

We performed a case-control study using the University of California Davis electronic medical records. The cases were defined as patients diagnosed with both psoriasis and hypertension, and controls were defined as patients with hypertension and without psoriasis. In this identified population, 835 cases were matched on age, sex, and body mass index (BMI) to 2418 control patients.

**Key Results:**

Treatment with multiple anti-hypertensives was significantly associated with the presence of psoriasis using univariate (p<0.0001) and multivariable analysis, after adjusting for diabetes, hyperlipidemia, and race (p<0.0001). Compared to hypertensive patients without psoriasis, psoriasis patients with hypertension were 5 times more likely to be on a monotherapy antihypertensive regimen (95% CI 3.607.05), 9.5 times more likely to be on dual antihypertensive therapy (95% CI 6.68–13.65), 16.5 times more likely to be on triple antihypertensive regimen (95% CI 11.01–24.84), and 19.9 times more likely to be on quadruple therapy or centrally-acting agent (95% CI 10.58–37.33) in multivariable analysis after adjusting for traditional cardiac risk factors.

**Conclusions:**

Psoriasis patients appear to have more difficult-to-control hypertension compared to non-psoriatic, hypertensive patients.

## Introduction

An expanding number of epidemiologic and immunology studies have provided new insight into the association between psoriasis and cardiovascular diseases [1]–[9]. The initial link between psoriasis and coronary artery disease was suggested in the 1970s [10]. Since then, multiple population-based studies have suggested an association between these two diseases [7], [11]–[13]. Results from a large population-based study showed an increased relative risk of myocardial infarction (MI) especially in young patients with severe psoriasis [1], [14], [15]. Psoriasis appears to be an independent risk factor for MI, even after controlling for traditional cardiac risk factors [4], [16], [17]. Additional population-based studies have supported a relationship between psoriasis and cerebrovascular and peripheral vascular diseases [2], [18].

Researchers have long suggested that vascular abnormalities might be present in patients with psoriasis [19]. Since the 1920s, histopathologists have observed the presence of abnormally dilated and tortuous capillaries in the dermal papillae and upper one-third of the dermis in psoriasis lesions [19]. The advent of electron microscopy enabled further characterization of these capillaries. These thin-walled vessels contained erythrocytes, displayed prominent gaps between endothelial cells, and lacked pericytes or surrounding smooth muscle cells [20]. Moreover, dilation of these abnormal capillaries was observed prior to keratinocyte hyperplasia in the early development of psoriasis lesions. Notably, the convoluted capillaries remained apparent in the upper dermis for many months even after cutaneous lesions had completely resolved [21], [22].

Previous large population studies have examined the relationship between psoriasis and hypertension and have focused on the frequency of hypertension occurrence in psoriasis patients compared to non-psoriasis patients [3], [23]–[25]. However, to our knowledge, hypertension severity (as measured by complexity of anti-hypertensive regimen) in psoriatic patients compared to non-psoriatic, hypertensive patients has not been described.

The primary aim of the study is to elucidate the relationship between the presence of psoriasis and severity of hypertension, as measured by number of anti-hypertensive classes used in each patient. Specifically, we sought to investigate whether patients with psoriasis have more severe hypertension or more difficult-to-manage hypertension compared to non-psoriatic hypertensive patients.

## Methods

Ethics Statement: This retrospective study was approved by the Institutional Review Board at University of California Davis (UCD). The Institutional Review Board at UCD approved a waiver of informed consent for this study.

In this case-control study, we defined “cases” as patients diagnosed with both psoriasis and hypertension and “controls” as those with hypertension and without psoriasis.

We searched UCD Health System EMR database from January 1, 2004 to July 5 2009 to identify our study cohort. We initially identified 1044 patients who had ICD-9 codes for both psoriasis and hypertension. From this initial cohort, we performed manual chart review, abstracting medical record individually to ensure that these patients fulfilled the following three criteria: (1) the diagnosis of psoriasis was given by a board-certified dermatologist, (2) the diagnosis of hypertension was made by the primary care physician, a cardiologist, or a nephrologist, and (3) the initial diagnosis of psoriasis was made either before or within 10 years after the diagnosis of hypertension. Of the 1044 patients identified as potential cases through EMR, we excluded 209 patients who did not meet the case definition. We included the remaining 835 patients as cases for the study—those having both psoriasis and hypertension. These cases were frequency-matched in a 1∶3 ratio to 2418 controls (non-psoriatic, hypertensive patients) on age, sex, and BMI. Inclusion in the control group required that hypertension was diagnosed by a primary care physician, cardiologist, or nephrologist.

The Seventh Report of the Joint National Committee on Prevention, Detection, Evaluation, and Treatment of High Blood Pressure (JNC 7) defines severity of hypertension based on the number and classes of antihypertensive agents [26]. Based on the JNC 7 criteria, we defined hypertension severity based on a patient's medication regimen: 0 = controlled with lifestyle modifications, 1 =  monotherapy (excluding centrally acting agents), 2 = dual therapy (excluding centrally acting agents), 3 = triple therapy (excluding centrally acting agents), 4 =  quadruple therapy and/or centrally acting agent.

We used anti-hypertension regimen as a measure for hypertension severity rather than discrete blood pressure measures because blood pressure measurements *alone* while on anti-hypertensive regimen have been considered a less reliable indicator of hypertension severity. We consulted with two board-certified University of California cardiologists to confirm that the use of anti-hypertensive regimen to define hypertension severity was applied adequately and accurately in our study. We also collected data on demographic and traditional cardiac risk factors in our study patients, including race, alcohol consumption, smoking status, and the presence of diabetes mellitus or hyperlipidemia.

Univariate and multivariate logistic regression analyses were performed. Factors that were significant on univariate analyses (p<0.05) were included in the multivariate logistic regression model. We also forced diabetes mellitus status into the multivariate model due to its clinical relevance. All statistical analyses were performed using SAS software, version 9.1 (SAS Institute). Two-tailed tests were used for all statistical analyses.

## Results

In this case-control study, the demographic and clinical factors for the cases (psoriatic, hypertensive patients) and controls (non-psoriatic, hypertension patients) are summarized in Table 1. Our study groups had a slight male predominance (53%), mean age of 62, and body mass index of approximately 31.

**Table 1:** Demographic and clinical characteristics of patients with psoriasis and hypertension (cases) and those with hypertension without psoriasis (controls).

Variable	Psoriasis & Hypertension (N = 835)		Hypertension (N = 2418)		P-value
	N	%	N	%	
Sex					
Female	390	47%	1130	47%	0.99‡
Male	445	53%	1288	53%	
Age					
Mean (±SD)	61.5±13.1 yr		61.7±12.8 yr		0.70*
BMI					
Mean (±SD)	31.0±6.7		30.7±6.2		0.28
Race					
White	319	38%	1120	46%	0.0058†
Black	22	2.6%	165	6.8%	
Asian	14	1.7%	80	3.3%	
American Indian	4	0.5%	7	0.29%	
Other	143	17%	540	22%	
Unspecified	333	40%	506	20.9%	
Smoking					

Percentages are followed by number of cases (in parentheses). For race, smoking status, and alcohol use, the analysis includes only patients with complete data on these parameters.

† Chi-square test was used to determine significance.

‡ Fisher's exact test was used to determine significance.

*Student's t-test was used to determine significance.

Our univariate analysis showed that the severity of hypertension, as defined in by the number of anti-hypertensives, was significantly associated with the presence of psoriasis (p<0.0001) (Table 2), and this relationship remains significant after adjusting for diabetes, hyperlipidemia, and race (p<0.0001) using a multivariable conditional logistic regression model (Table 3).

**Table 2:** Univariate analysis examining relationship between hypertension severity and presence of psoriasis.

Variable	Cases				Controls			Crude OR	95% CI	P-value
	N		%		N		%			
Sex										
Female	390	47%		1130		47%		–	–	–
Male	445	53%		1288		53%		1.00	0.86-1.17	0.99
Race										
White	319	64%		1120		59%		–	–	–
Black	22	4%		165		9%		0.47	0.30–0.74	0.0013
Asian	14	3%		80		4%		0.61	0.34–1.10	0.10
American Indian	4	0.80%		7		0.37%		2.01	0.58–6.90	0.27
Other	143	28%		540		28%		0.93	0.74–1.16	0.52
Smoking										
Never	382	47%		1093		52%		–	–	–
Prior Smoking	310	39%		745		36%		1.59	1.10–1.58	0.0025
Active Smoking	113	14%		260		12%		1.38	1.07–1.77	0.01
Alcohol Use										
No	308	41%		925		45%		–	–	–
Yes	441	59%		1119		55%		1.18	0.10–1.40	0.05
Type II Diabetes Mellitus										
No	381	46%		1181		49%		–	–	–
Yes	454	54%		1237		51%		1.14	0.97–1.33	0.11
Hypertension										
Lifestyle Modification	113	14%		1248		54%		–	–	–
Monotherapy	299	36%		728		30%		4.54	3.59–5.74	<0.001
Dual Therapy	239	29%		301		12%		8.77	6.78–11.34	<0.001
Triple Therapy	148	18%		116		4.80		12.09	10.33–19.21	<0.001
Quadruple or Central Acting Therapy	36	4.30%		25		1%		15.90	9.22–27.44	<0.001

**Table 3:** Multivariate analysis examining relationship between hypertension severity and presence of psoriasis adjusting for diabetes mellitus, smoking, hyperlipidemia, and race.

Variable	Adjusted OR	95% CI	P-value
Race			
White	–	–	–
Black	0.36	0.21–0.60	<.001
Asian	0.66	0.34–1.25	0.20
American Indian	0.87	0.17–4.45	0.86
Other	1.03	0.80–1.32	0.83
Smoking			
Never	–	–	–
Prior Smoking	1216	0.95–1.55	0.11
Active Smoking	1.43	1.01–2.02	0.04
Type II Diabetes Mellitus			
No	–	–	–
Yes	1.07	0.83–1.37	0.62
Hyperlipidemia			
No	–	–	–
Yes	1.15	0.92–1.45	0.22
Hypertension			
Lifestyle Modification	–	–	–
Monotherapy	5.04	3.60–7.05	<.001
Dual Therapy	9.55	6.68–13.65	<.001
Triple Therapy	16.54	11.01–24.84	<.001
Quadruple or Central Acting Therapy	19.87	10.58–37.33	<.001

Controls are age, sex, and BMI–matched.

Compared to hypertensive patients without psoriasis, psoriasis patients with hypertension were 5 times more likely by multivariate analysis to be on monotherapy antihypertensive regimen (95% CI 3.60–7.05), 9.5 times more likely to be on dual antihypertensive therapy (95% CI 6.68–13.65), 16.5 times more likely to be on triple antihypertensive regimen (95% CI 11.01–24.84), and 19.9 times more likely to be on quadruple therapy or centrally-acting agent (95% CI 10.58–37.33), after adjusting for diabetes, hyperlipidemia, smoking status, and race. These results suggest a significant correlation between the treatment-refractory hypertension and presence of psoriasis that is independent of other known cardiovascular risk factors.

## Discussion

The precise pathophysiologic mechanisms that underlie psoriasis and hypertension are unknown. Researchers proposed that adipose tissue in psoriasis patients serves as a major source of angiotensinogen, which is subsequently converted to angiotensin II [27]. Angiotensin II not only promotes salt retention by kidneys; it also stimulates T-cell proliferation [28], [29]. Angiotensin II also appears to promote inflammation and the development of atherosclerosis [30]. The association between psoriasis and hypertension may also be attributed to the increased oxidative stress in psoriasis patients [31]. Greater levels of reactive oxygen species can damage endothelium-dependent vasodilation.

Some researchers have suggested that increased visceral adipose tissue in psoriasis patients may contribute to hypertension development. Increased visceral adipose tissue may be associated with accumulation of perivascular fat, which can serve as a reservoir for activated effector T cells that promote dysfunction in both hypertension and psoriasis [1]. However, these findings do not entirely explain the persistent, significant association between psoriasis and hypertension after adjustments for BMI.

Other investigators suggest that endothelin-1 may play an important role in the development of hypertension among psoriasis patients. Endothelin-1 is a protein that constricts blood vessels and increase blood pressure, and it is produced by several different cell types including keratinocytes. While the level of endothelin-1 is usually regulated through various mechanisms, their expression appears to be altered in psoriasis patients [32]. Compared to individuals without psoriasis, the level of endothelin-1 appears to be increased in lesional skin and the serum of psoriasis patients. Furthermore, endothelin-1 level appears to correlate with psoriasis disease severity [32]. Increased endothelin-1 levels are thought to exert a greater vasoconstrictive effect on the blood vessels, which contributes to the development of hypertension.

Previous population studies comparing psoriasis patients with the general population have shown a modest increase in the relative risk of developing hypertension (Table 4) [3], [23], [25], [33]. Specifically, in a prospective study among U.S. female nurses, Qureshi et al. found that women with psoriasis experienced an increased risk for developing hypertension (RR 1.17; 95% CI 1.06–1.30) [3]. In a case-control study using a health-maintenance organization database, investigators found modestly increased odds of having hypertension among psoriasis patients (OR1.37, 95% CI: 1.29–1.46) [23]. Most previous population studies have relied solely on ICD-9 diagnostic codes to identify patients with psoriasis. However, due to potential misclassification errors by providers or coders, not all ICD-9-based diagnoses in the EMR or claims databases reflect actual diagnoses of medical conditions, including psoriasis or hypertension. Therefore, we employed the method of reviewing individual physician notes to ascertain whether patients had physician-diagnosed hypertension or psoriasis.

**Table 4:** Studies of prevalence of hypertension among psoriasis patients.

Author and Journal	Study Design	Number of Psoriasis Patients	Prevalence of Hypertension	P-value
Armstrong, et al. 2010 (Current study)	Case-Control	835	See details in text	See details in text
Al Matari, et al. 2010*Japanese Dermatological Association*	Case-Control -computerized patient records	Mild-Moderate - 1661	OR = 3.597(95% CI 3.015–4.29)	<0.001
		Severe - 129	OR = 5.17(95% CI 3.53–7.55)	
Altobelli, et al. 2009*European Journal of Dermatology*	Survey - questionnaires	Total - 1376	Frequency of associated HTN = 12.9%	<0.001
		Smokers (>15 cigarettes per day)	OR = 1.37(95% CI 1.01–2.03)	
		Drinkers (>2 glasses/day of wine)	OR = 2.11(95% CI 1.31–3.40)	
Neimann, et al. 2009*Journal of the American Academy of Dermatology*	Cross-Sectional Study - UK General Practice Research Database	Mild – 127,706	OR = 1.16(95% CI 1.14–1.18)	95% CI
		Severe – 3,854	OR = 1.25(95% CI 1.13–1.39)	
Qureshi, et al. 2009*Archives of Dermatology*	Cohort - women aged 27–44 in 1991, completed follow-up questionnaire in 2005	78,061 out of the 116671 from the 1991 cohort responded in 2005	Age Adjusted RR = 1.32(95% CI 1.19–1.45)Multivariate RR = 1.17(95% CI 1.06–1.30)	95% CI
Cohen, et al. 2010*Acta Derm Venereol*	Case-Control – Clalit Health Services Database	12,502	OR = 1.37(95% CI 1.29–1.46)	<0.001
Augustine, et al. 2010*ActaDerm Venerol*	Case-Control – Health Insurance Data in Germany using ICD-10 codes	33,981	OR = 1.73(95% CI 1.71–1.76)	N/D
Wu, et al. 2008*Journal of Drugs in Dermatology*	Case Control - National Health and Wellness Survey	1127	1.49(95% CI 1.23–1.80)	<0.01
Kaye, Li, and Jick 2008*British Journal of Dermatology*	Cohort – General Practice Research Database	44,164	HR = 1.09(95% CI 1.05–1.14)	<0.001
Sommer, et al. 2006*Arch Dermatol Res*	Case-Control	581	OR 3.27(95% CI 2.41–4.43)	<0.001

In this study, we sought to address whether psoriasis patients have more difficult-to-control hypertension compared to hypertensive patients without psoriasis. By defining cases as patients with both psoriasis and hypertension and controls as patients with hypertension alone, we ascertained that psoriasis patients were more likely to require more anti-hypertensive medications compared to those without psoriasis on both univariate and multivariate analyses after adjusting for potential confounders. These findings suggest that patients with psoriasis tend to have required a greater number of anti-hypertensive classes prescribed in these patients.

The findings of this study need to be interpreted in the context of the study design. In this case-control study, the medical records for the cases and the controls have been individually evaluated to ensure that they represent true cases or controls. This method of defining the cases and controls, though time-consuming, allowed for more accurate identification of the comparison groups. While this research methodology does not completely eliminate misclassification errors, it greatly reduces the likelihood of misclassification of cases or controls, a problem frequently encountered when using ICD-9 codes alone in population studies. Despite our best efforts to minimize misclassification errors, it is still possible that a patient with potentially severe hypertension was not on multiple anti-hypertensive medications due to not obtaining regular medical care. Although the current medical literature remains inconclusive at best regarding whether certain anti-hypertensive medications exacerbate psoriasis, it is nevertheless important to consider this possibility in a small number of patients. While we considered assessing psoriasis severity, such assessment is hampered by the lack of consistent PASI or body surface area recordings. Furthermore, in comparison to hypertension that follows a more predictive, progressive course, the clinical course for psoriasis as measured by medications is much more variable over several years. Therefore, the fluctuating nature of psoriasis disease severity makes it potentially problematic to select one data point to define the overall disease severity in a patient.

Our findings contribute to the current literature by elucidating the relationship between psoriasis and hypertension. While previous studies have shown modestly increased odds of having hypertension among psoriasis patients, our study findings suggest that psoriasis patients who have hypertension are more likely to have more difficult-to-control hypertension and require a greater number of anti-hypertensive medications than non-psoriatic, hypertensive patients. These novel findings will contribute to our understanding of the epidemiologic associations between these two conditions and provide impetus for in-depth translational and basic investigations that elucidate shared mechanisms for this epidemiologic observation. Furthermore, the results of this study may benefit physicians who regularly treat hypertensive patients and alert them to the likelihood that the psoriatic patients will likely require more intense hypertensive regimen to achieve adequate blood pressure control, compared to other hypertensive patients.
